# What Makes a Great Peer Reviewer in Neurology Education?

**DOI:** 10.1212/NE9.0000000000200139

**Published:** 2024-06-19

**Authors:** Roy E. Strowd

**Affiliations:** From the Department of Neurology, Wake Forest University School of Medicine, Winston Salem, NC.

Peer reviewers are the engine that drive much of academic scholarship forward. For most peer reviewers, their work is uncompensated, driven by a passion for scientific advancement, and essentially academic volunteerism. High-quality peer reviewers who are consistent, timely, and constructive in their review are an editor's best friend and frequent confidant. With this in mind, I cannot express the depth of gratitude for all of the outstanding reviewers who have contributed to *Neurology*® *Education* over the past year. They deserve considerable recognition and great thanks for advancing the scholarly work in *Neurology: Education*.

## Thank You to Our Peer Reviewers

Since launching the Call for Peer Reviewers over a year ago, we have received over 110 requests to contribute to the journal as a peer reviewer. The reviewer pool now includes a diverse cohort of neurologists, non-neurologists, methodological experts, teaching scholars, as well as trainees who provide an important perspective on the impact and relevance of educational interventions from the perspective of the learner. *Neurology: Education* remains committed to disseminating the highest quality, evidence-based training that advances teaching and learning in the clinical neurosciences. This requires considerable time and effort from our peer reviewers. We cannot thank you enough.

## What Is the Importance of Peer Review in Academic Publishing?

Peer review has become a cornerstone of scientific publishing. Reviewers serve as more than a mere gatekeeper for quality literature. Their comments and critiques enhance research quality, advance the integrity of reporting, promote rigor and reproducibility of results, and propel the field forward. The critical role of peer review has become even more evident with the proliferation of misinformation, concerns over reproducibility, demand for expedited publication, and rise of artificial intelligence (AI) facilitated by open AI platforms.

Despite the vital nature of peer review in academic neurology, formal and even informal training in how to conduct a peer review and when to begin this part of academic service is notably absent from most training pathways in clinical medicine. Residents, junior neurologists, and even midcareer faculty are often forced to develop these skills and navigate this world using informal mentoring and a trial-and-error approach.

## What Does It Take to Be a Great Peer Reviewer?

To effectively evaluate a manuscript, a reviewer must first have content expertise. This is a necessary but not sufficient prerequisite for peer review. There are also other critical skills that high-quality reviewers possess (Figure). These can take a good critique and formulate it into a great review. In addition to content expertise, these reviewers often possess critical appraisal skills, strong written communication skills, and a working understanding of publication science and journalology.^1^ Each of these is a critical component to being a well-rounded, high-quality reviewer, and each can be obtained in several different ways.

**Figure:**
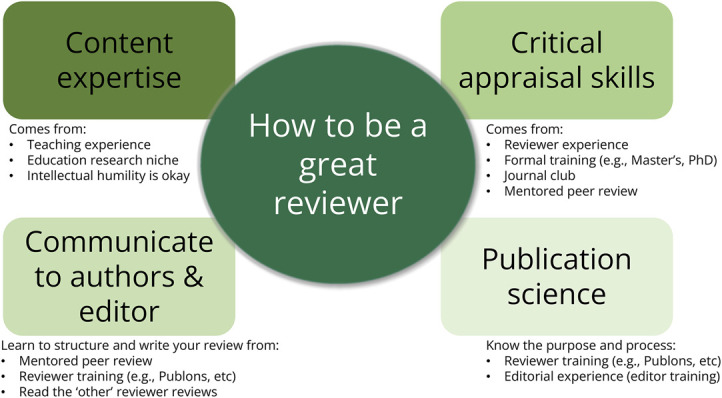
Key Competencies to Be a Great Reviewer

### Content Expertise

To effectively contribute as a reviewer, one must possess a robust foundation of content expertise. This requires not only having the knowledge to comprehensively grasp the educational topic but also the experience in teaching to contextualize and evaluate their significance. Reviewers must draw on this knowledge and experience to determine whether the intervention is in fact important, relevant, impactful, and rigorously conducted. For many reviewers, content expertise is gained over time by reading, teaching, writing, and engaging in evidence-based education. This means that the reviewer should have adequate knowledge to understand the teaching gap, enough educational experience to put that gap into context, and at times sufficient intellectual humility to recognize the limits of one's knowledge and dive into a mini literature review, reading related articles, and explore cited references when reading a manuscript. A bit of imposter syndrome can be a good thing.

### Critical Appraisal Skills

Critical appraisal is arguably one of the most important skills in separating good from great reviews. These reviewers can take a complex manuscript and break it down into its constituent parts. This requires a nuanced understanding of education research methodology and often an ability to understand the conceptual model or theoretical framework that undergirds the educational intervention. Manuscripts should clearly define the problem statement and describe the gap that exists in teaching practice. The methods should be clearly described, reproducible, and they should convey the primary learner objectives and how the outcomes were measured. The results should demonstrate achievement of these outcomes in the same order and number. Lessons learned should be clearly articulated in a way that other educators can adopt. Each of these requires honing critical thinking skills. Formal training (e.g., Master's program, Doctor of philosophy), journal clubs, and mentored peer reviewing can be some of the many ways to cultivate such an approach to critical appraisal.

### Scholarly Communication Skills

Effective communication with authors and editors is a cornerstone of peer review. Reviewers are communicating to many groups, often all at once. This includes the authors, handling editor, editor-in-chief and at times a statistical or methodological expert, diversity/equity/inclusion expert, and others. It is not enough to simply recognize deficiencies in a manuscript and areas that could be improved. It is imperative that this be communicated in a way that will be easily understood and achievable by the authors. This proficiency often stems from mentored peer review experiences. Reviewers can often read the review critique from the other reviewers of a manuscript, which is a great way to continuously learn and refine one's language and approach.

### Understanding Publication Science

Finally, some reviewers will find it incredibly valuable to understand how a journal works from the back end. This includes understanding the types of peer review (i.e., open peer review), the types of reviewer and author blinding, the purpose of confidential comments to the editor and open comments to the authors, as well as what drives decision-making. In general, all critiques about a manuscript should be disclosed to the authors. The comments to editor are a place to explain the reason for the reviewer's recommendation and disclose any perceived conflicts of interest or publication ethics concerns. Increasingly, online training curricula are available to help demystify the publication process and open the publication hood to provide reviewers with a more in-depth understanding of the review process.

As we take time to thank our many committed reviewers from the last year, I look with great optimism to the coming year where we will continue to expand the pool of high-quality reviewers for *Neurology: Education*. As lifelong learners, we all can continue to hone these four reviewer skill areas: content expertise, critical appraisal skills, scholarly communication abilities, and an understanding of publication science.

The future is bright for *Neurology: Education*. Happy reading.

The below list includes those reviewers who returned a review or reviews of initial submissions between January 1, 2023, and December 31, 2023. Reviewers with 1 asterisk have reviewed 5 or more articles while 2 asterisks indicate that the reviewer has reviewed 10 or more articles.

Paula Adler*

Dara V.F. Albert**

Catherine Albin

Megan C. Alcauskas

Abeer F. Almarzouki

Essam M. Al-Sibahee

Corrado I. Angelini

Aileen A. Antonio*

Farzaneh Barzkar

Michelle Bell

Diana Benea

Aaron L. Berkowitz

Catarina Bernardes

Miya E. Bernson-Leung*

Andrea Berry

Gundula Bosch*

Richard B. Carozza

Joseph Carrera

Nikita Chhabra*

Nicole Ann Chiota-Mccollum**

Shilpa Chitnis*

Elizabeth A. Coon*

Claire J. Creutzfeldt

Cole Crowson*

Saurav Das*

Paul De Roos*

Kathryn E. Dent

Jeffrey Dewey

Daniel G. Di Luca*

Asif Doja

Christopher Doughty

Cheran Elangovan

Amtul S. Farheen

Renato Faria da Gama

Andres Fernandez*

Jenna Ford*

Mark Friedman

Steven L. Galetta*

Joseph R. Geraghty

Shivani Ghoshal

Chris Gillette*

Larry B. Goldstein

Jerome J. Graber*

Patricia Jokl Graese

David Matthew Greer

Robert A. Gross

Scott N. Grossman

Preeta Gupta**

Deepak K. Gupta

Amy Katherine Guzik

Faizal Aminmohamed Haji*

Roy H. Hamilton

Daniel S. Harrison

H.E. Hinson

Jennifer L. Hopp

Brendan Huang

Nuri Jacoby

Dennis Keselman

Pearce J. Korb**

Natalie A. Kukulka

Douglas P. Larsen*

Steven M. Lazar**

Hernan Nicolas Lemus

Ruoxuan Li

Zachary London*

Marie Charmaine Sy Lukban**

Tomoyasu Matsubara

Mark J. Milstein

Jeremy J. Moeller**

Kathryn Moore

Daniel Moreno-Zambrano

Solomon L. Moshe

Fábio Augusto Nascimento

Kathryn Nevel

Sunjay Parmar

Jorge Patino

Gustavo A. Patino

Pritikanta Paul

Gauri V. Pawar

Elizabeth Pickup

Ana Ponciano

Subha Ramani*

Mehmood Rashid*

Jeffrey B. Ratliff

Matthew Stuart Robbins*

Kasser Saba

Rana R. Said*

Rachel Marie E. Salas*

Simone Salemme

Stefano Sandrone**

Sara M. Schaefer

Suma Shah

Madhu Soni*

Marinos G. Sotiropoulos

Andrew M. Southerland

Stephen M. Sozio

Michael P.H. Stanley

Kara A. Stavros

Robert Thompson Stone

Isabella Isabella Strozzi

Eric Stulberg

Jimmy Suh

Harry W. Sutherland

Christopher Tarolli

Anna R. Thamann

Edward H. Yu

Aaron S. Zelikovich

Allison Jing Zhong
